# Organic Consumer Choices for Nutrient Labels on Dried Strawberries among Different Health Attitude Segments in Norway, Romania, and Turkey

**DOI:** 10.3390/nu11122951

**Published:** 2019-12-04

**Authors:** Valérie L. Almli, Daniele Asioli, Celia Rocha

**Affiliations:** 1 Department of Innovation, Sensory and Consumer Sciences, Nofima AS, NO-1431 Ås, Norway; valerie.almli@nofima.no; 2 Department of Applied Economics and Marketing, School of Agriculture, Policy and Development, University of Reading, Reading RG6 6AR, UK; 3 GreenUPorto—Sustainable Agrifood Production Research Centre & LAQV-REQUIMTE/DGAOT, Faculty of Sciences, University of Porto, Edifício das Ciências Agrárias (FCV2), 4485-646 Vila do Conde, Portugal; crocha@fc.up.pt; 4 Sense Test, Lda., 4400-345 Vila Nova de Gaia, Portugal

**Keywords:** organic consumer choices, organic dried strawberries, nutrient labels, health attitudes, consumer heterogeneity, cross-cultural comparison

## Abstract

Consumer interest towards healthy food is driving the growth of the organic food market because consumers perceive organic food products to improve their personal health. Berries have well-known health benefits and show increasing market shares in European markets. This manuscript investigates for the first time how health attitudes relate to organic consumers’ choices for nutrient labels of organic dried strawberry products. We conducted an online survey with 614 consumers from Norway, Romania, and Turkey. All participants consumed and liked strawberries and purchased organic food at least once a month. Participants filled out attitudinal questionnaires and conducted an experimental choice task featuring paired images of packaged organic dried strawberries varying in nutrients content label and other factors. The pooled sample was split into three groups of varying health attitudes for profiling and choice analysis. The results show that broad variations exist in health attitudes among Norwegian, Romanian, and Turkish organic consumers. A non-linear effect of health attitude is revealed, where a moderate health attitude is more strongly associated with the selection of products with increased nutrients content than either a low or a high health attitude. The results highlight the complexity in targeting nutrition labels to organic consumers. Finally, implications and suggestions for organic food operators are discussed along with future research avenues.

## 1. Introduction

Diet-related health problems evidenced by the increasing number of food-linked diseases as well as the consumer concerns towards the growing industrialized food production have dramatically increased over the last decades [1,2,3,4,5]. This has raised the importance of nutritional information as a relevant criterion affecting consumers’ food choices [1]. Indeed, one of the most important trends that is affecting food consumption is the increasing consumer interest towards food products that provide health benefits [6,7,8]. This growing interest is also signaled by the increasing number of food products on the market that feature health claims or other labels that consumers infer as beneficial to health (e.g., functional foods, organic products, clean labels, or local food) [9]. A typical way to convey information about the healthiness of food products is through the back-of-pack nutrition label [4,10,11]. Such nutrition labels are an important tool that food marketers can use to communicate information about the nutritional value and composition of the products and at the same time they are a valuable tool to help consumers make informed decisions [11]. Yet studies have shown that few consumers read nutrition labels [12], and marketers as well as governments are prone to use front-of-pack labels to highlight specific nutritional features [13,14].

Berries have a well-known balanced nutritional composition and healthiness properties due to their richness in vitamins, fibers, antioxidants, polyphenols, and minerals that consumers appreciate as part of a healthy diet [15]. Thus, berries are suggested to potentially reduce the risk of cardiovascular diseases, inflammation, obesity, diabetes, cancer, and other chronic diseases [16,17,18]. Strawberries in particular are among the most popular and widespread berries on the food market [19] as their sensory [20,21] and nutritional properties [22] are highly appreciated by consumers [23,24]. However fresh berries are highly perishable and strawberries typically have a shelf life of approximately one week only, assuming chilled storage conditions (typically 6 °C or less depending on the variety) [25]. Several strategies can be used in order to increase shelf life of strawberries, including antimicrobial edible coating, gamma-irradiation, modified atmosphere packaging and antimicrobial active packaging of fresh berries [26,27,28,29,30], transformation into puree or juice possibly combined with high pressure and thermal processing [31], or drying [32,33,34]. A conventional drying process such as convective air-drying allows to extend shelf life considerably, but not without loss of nutritional and sensory qualities of the berry [35]. Recent mild processing technologies (e.g., microwave-vacuum) are able to dry the berries while preserving their nutritional quality by retaining more of the initial ascorbic acid, anthocyanins and phenolic compounds, and antioxidant capacity [36,37,38].

Organic foods are a product category that consumers perceive to be healthy [39,40,41]. Indeed, a large number of studies show that organic consumers’ choices are mainly driven by the perceived naturalness and healthiness of these products compared to conventional food products [41,42,43,44,45,46,47,48]. The European organic food market is currently one of the most rapidly expanding food markets (+114% between 2006 and 2017) [49], and Europe represents the second largest organic agricultural area in the world (13.5 million hectares) after Oceania (27.3 million hectares) [50]. However, large discrepancies exist within Europe and it is therefore important to consider multicultural studies when it comes to the European organic consumer. In particular, in 2016 the organic food market retail sales weighed 394 million Euros in Norway (population: 5.2 million inhabitants), with 4.8% of total agricultural land dedicated to organic production [50]. In contrast, the organic market in Romania (population: 19.6 millions) represented 80 million Euros (Data from 2011) with 1.7% of agricultural land dedicated to organic production, and 4 million Euros in Turkey (Data from 2009) (population: 82 millions) with 1.4% of organic land [50]. These three countries exemplify therefore different maturity levels of the organic market in Europe.

This manuscript investigates organic consumers’ choices for nutrient labels of dried strawberries based on their health attitude. While a large number of studies have investigated consumers’ preferences and willingness to pay (WTP) for organic food products [51,52,53] and explored drivers of consumption [47,54,55,56,57], personal determinants [58,59], subjective norms and attitudes [58], sensory attributes [44,60,61,62,63], and organic labeling [64], no prior study has investigated their preferences for nutrient labels in relation to their health attitudes. Eagly and Chaiken [65] define attitude as “a psychological tendency that is expressed by evaluating a particular entity with some degree of favor or disfavor”. In practice, attitudes are normative, valuative, subjective and may expand to opinion, habits, tendencies to act, impulses to act, inhibitive impulses, feelings, wishes, and values [66]. Attitudes are learned and not inherited [67]. Besides forming from experience, they also derive from social influence, where social attitudes define a group of similar status in a specific domain [67]. Further, Hong [68] presents health consciousness as an “important psychographic variable (…) [that] predicts a variety of health attitudes and behaviors” and “is closely related to how [an individual] seeks and responds to health information”. According to this author, health consciousness is a five-dimensional concept related to integration of health behavior, attention to one’s health, health information seeking and usage, personal health responsibility, and health motivation.

The relationship between organic consumers and health has been investigated by several authors. While health consciousness has been found to predict attitudes and intention of purchasing organic foods [69,70], the relationships between health consciousness, attitudes, and consumers’ preferences are ambiguous. Indeed, Michaelidou and Hassan [71] found that health consciousness is the least important motive in predicting attitudes towards organic produce while Magnusson et al. [70] indicated that it is a motive for shaping attitude towards organic produce. In addition, Hansen, Sørensen, and Eriksen [41] reported that health consciousness has a high positive influence on organic food identity. It is therefore not clear if and how different health attitudes may drive organic consumers’ preferences towards nutrition labels. This information may help organic-food marketers to better target nutrition labeling strategies at consumer segments of different health attitudes.

Our study aims to highlight the heterogeneity in health attitudes among multicultural organic consumers, and establish possible relationships between health attitudes and consumers’ attractiveness for nutrition labels. New mild processing technologies may better retain the original nutrients content in organic products such as dried strawberries—our underlying question is: will organic consumers be attracted to more nutrients, and is that attractiveness related to their health attitude? We utilize data from Norway, Romania, and Turkey, where consumers’ choices for organic dried strawberry products of varying nutrients labels as well attitudinal, behavioral, and socio-demographic characteristics were collected. We profile three consumer groups of low, medium, and high health attitudes in terms of attitudinal, behavioral, and socio-demographic characteristics. We then model consumer choices for organic dried strawberry products labeled with “more natural nutrients” versus “natural nutrients” labels with regard to health attitude groups. The results show that broad differences exist in terms of health attitude among European organic consumers, and that health attitude has a non-linear relationship to the selection of products with increased nutrients content. We discuss the results in light of previous literature and conclude in terms of implications for the promotion of nutritional foods to organic consumers.

## 2. Materials and Methods

An online consumer survey was conducted in the Winter of 2017 in the three target countries (i.e., Norway, Romania, and Turkey). Respondents completed a choice task for dried strawberry properties and responded to attitudinal, behavioral, and socio-demographic questions.

### 2.1. Choice Task

**Figure 1:**
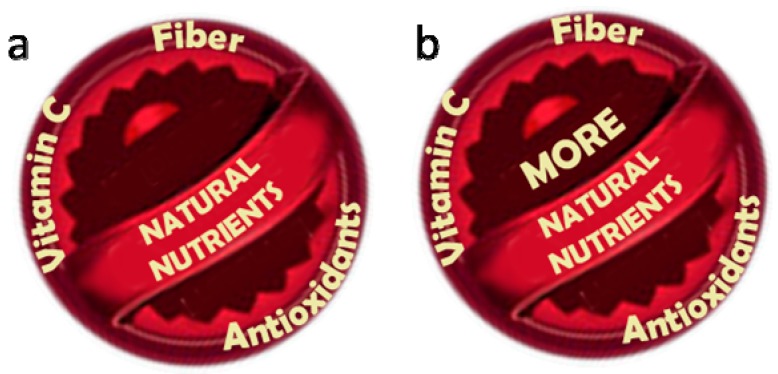
“Natural nutrients” (**a**) and “More natural nutrients” (**b**) labels utilized on organic dried strawberry packages in the experimental choice task (English translation).

Consumers participated in an online choice experiment where several mock-up images of dried strawberry packages were used. All packages included an organic logo. The images varied in nutrients labels, as nutrients is one of the key attributes that consumers consider when purchasing organic foods [72] and berry products [21,73]. Two levels of nutrient content were specified: “Natural nutrients (Antioxidants, Vitamin C and Fibres)” or “More natural nutrients (Antioxidants, Vitamin C and Fibres)”. Except for the word ‘more’, the two labels were kept identical with regards to size, fonts, colors, and remaining text in order to elicit consumer responses to nutrients content and nothing else (Figure 1). In addition to nutrients, the dried strawberry packages varied in origin (Europe or own country), technology (air drying or microwave drying) and price (three levels varying from 10% to +10% of the average market prices in each target country) following a D-optimal choice design of 11 choice sets. Each choice set was composed of two product alternatives (options A and B) and an ‘opt-out’ option (option C) (see an example in Supplementary Materials Figure S1). To make sure that respondents would notice and understand the different product attributes, an introduction screen presented one of the mock-ups and the four attributes.

In the present paper, focus is brought on consumer choices for products labeled with “more natural nutrients” as opposed to “natural nutrients”. For more details on the choice design and analysis of consumer preferences with regard to all four attributes, see Asioli et al. [74].

### 2.2. Attitudinal and Behavioral Questionnaire

After the choice task, we included in the survey selected attitudinal question items related to consumers’ health (HAQ) and natural (NAQ) attitudes selected from the Health and Taste Attitude Questionnaire [75], consumers’ ecological attitudes using the Food-Related Lifestyle (FRL) scale [76] and consumers’ attitude towards new food technology using the Food Technology Neophobia Scale (FTNS) [77] (Table 1). All attitudinal questions were collected using scales anchored from 1 (strongly disagree) to 7 (strongly agree). Furthermore, the questionnaire included a set of behavioral items with regards to purchase and consumption habits of strawberry products (i.e., purchase frequency, point of sale, search criteria) (Table 1). The survey development was executed in English, then translated in Norwegian, Romanian, and Turkish by the local research teams, then carefully checked against the original English questionnaire. The global survey was pretested by 6 to 19 respondents (colleagues of the local teams) in each country during Fall 2016. After adjustments, the survey was pilot-tested (*n* = 20 per country), before the full data collection took place in February 2017. The full questionnaire is available upon request.

### 2.3. Data Collection

The data were collected during Winter 2017 involving a total of 614 consumers in Norway (*N* = 204), Romania (*N* = 206), and Turkey (*N* = 204) using the online platform Userneeds (Copenhagen, Denmark). Consumers where randomly recruited by Userneeds using sampling quotas in terms of age and gender. Participants were informed about the opportunity to participate in a survey on consumers’ valuation of berry products. An overall sample of 13,070 consumers were invited by e-mail to participate to the web-survey, wherein 614 consumers (4.7% of the invited consumers) fulfilled the eligibility criteria and completed the survey. We recruited only consumers aged between 18 and 65 years, who consume and like strawberries, purchase organic foods once a month or more often (“I purchase organic food products… At least once a week/At least once a month/A few times a year/Rarely or never”), and had purchased and consumed dried nuts, fruits and/or berries at least once during the last three months prior to the survey. Dried strawberries is not a common product as such in the three surveyed countries to date, but may be encountered in, e.g., breakfast cereals. We recruited only consumers who eat and like strawberries and purchased dried fruits, dried berries and/or nuts in the last three months, as these may represent potential customers of dried strawberries if the product expands on the market. Respondent age groups were equally balanced (18–29 years old: 25.9%, 30–41 y.o.: 25.9%, 42–53 y.o.: 23.1%, 54–65 y.o.: 25.1%), 50.2% of the respondents were males, and 79.1% of the pooled sample reported a university education or equivalent. These sociodemographic characteristics were similarly distributed across the three countries. For further details about the sociodemographic characteristics of the sample investigated, see [74]. During the test, the participants first received the recruitment questionnaire. Eligible participants proceeded to the description of the attributes and levels in an introductory screen, then to the choice task. Upon completion of the series of choice tasks, they filled out the attitudinal, behavioral, and socio-demographic questionnaire. The survey took approximately 8–10 min to answer. The data were collected with compliance to the ethical principles of the declaration of Helsinki.

### 2.4. Data Modeling

#### 2.4.1. Attitudinal Groups

To start with, individual mean attitudinal scores were derived from the HAQ, NAQ, FRL, and FTNS scales by summing up the respondents’ answer scores across the different items within each scale. Negatively-phrased question items were reversed before summing (Table 2). Furthermore, in order to develop models for different levels of health attitude, three consumer groups (HAQ1, HAQ2, and HAQ3) were defined cross-nationally based on question items for HAQ by using the 25% and 75% quartiles as cut-off thresholds. Also attitudinal groups related to NAQ (two groups split at median), FRL (two groups split at median), and FTNS (two groups split at median) were defined in order to profile our health attitude groups in relation to natural, organic and technology attitudes. It should be noted that all attitudinal variables presented nearly normal distributions (skewness HAQ: 0.15, NAQ: −0.38, FRL: −0.50, FTNS: 0.08). The group splits are therefore arbitrary and only defined to highlight different effects between lower/higher attitudinal scores in the respondent sample in further analyses.

#### 2.4.2. Characterization of Health Attitude Groups with Partial Least Squares Regression (PLS-R)

We related the respondents’ variations in health attitude levels to socio-demographic characteristics, other attitudinal dimensions (i.e., NAQ, FRL, and FTNS scores) and prioritized factors in the choice task (i.e., individual coefficients from the ML model). A partial least squares regression (PLS-R) was used for the purpose as it is able to handle highly correlated variables [78]. The base model included all socio-demographics and questionnaire variables as independent variables (X) and HAQ as the dependent variable (Y). Both models utilizing HAQ as a continuous variable as well as models utilizing HAQ1, HAQ2, and HAQ3 as binary variables coding for group belonging were attempted. In all models, cross-validation with 20 random segments and uncertainty testing based on jack-knifing (95% confidence interval) were used for model validation and variable selection [79]. Only the refined models for HAQ1 and HAQ3 are presented in the results section as these best identified respondent characteristics associated to variations in health attitude levels. The models were run in The Unscrambler X 10.4.1 (Camo Software AS, Oslo, Norway).

#### 2.4.3. Individual Choices for “More Nutrients”

The data collected in the choice tasks were analyzed using the mixed logit (ML) model [80], estimating the utility of each product alternative for each individual in each choice occasion. This model is based on all 11 choice sets, and includes main effects and two-way interactions of the attributes origin, technology, nutrition content, and price. Full details on this model and its results are given in [74] and will not be discussed here. For the purpose of this study, individual parameter estimates (beta coefficients) for attribute nutrients were then extracted from the ML model and utilized as a measure of consumers’ nutrient label preferences in further analyses. This approach allows focusing on attribute nutrients alone in the present report while accounting for the impact of the other attributes during the choice experiment. The ML model was estimated using the Stata module *mixlogit* while the individual parameter estimates were extracted using the Stata module *mixlbeta* run in STATA 15.0 software (StataCorp LP, College Station, TX, USA).

#### 2.4.4. Analysis of Variance (ANOVA)

We utilized analysis of variance (ANOVA) to investigate socio-demographic and attitudinal effects related to respondents’ selection of organic dried strawberry products with either standard or increased nutrients contents in the choice task. A first ANOVA was conducted in a general linear model with factors country (three levels), gender (two levels), age (four levels: 18–29; 30–41; 42–53; and 54–65 years), organic consumption frequency (two levels), HAQ (three levels), NAQ (two levels), FRL (two levels), FTNS (two levels), income (four levels), urban area (two levels), and university (two levels) as well as their two-way interactions. Individual ML model estimates for design factor “nutrients” were used as a dependent variable. The primary ANOVA model was refined in a second model by withdrawing non-significant main effects that were not involved in interactions and non-significant interactions (*p*-value > 0.05). Only the final model is presented in the results section, which includes the factors country, gender, age, HAQ, FTNS, income, urban area, university and selected two-way interactions. ANOVA models were run in Minitab 18.1 (Minitab, Inc., State College, PA, USA).

## 3. Results

### 3.1. Attitudinal Characteristics in Norway, Romania, and Turkey

Table 2 reports descriptive statistics (i.e., mean and standard deviations) of the attitudinal questions computed across the three countries. The results show that Turkish and Romanian organic consumers have significantly larger pro-health and pro-natural product interest compared to Norwegian consumers, and in turn Romanian consumers have significantly larger pro-ecological attitudes compared to Turkish and Norwegian consumers. Romanian organic consumers also show a significantly higher food technology neophobia compared to Turkish and Norwegian consumers, and in turn Turkish consumers have significantly higher food technology neophobia compared to Norwegians. Table 2 also reports the group thresholds used for further data analysis of attitudinal variables (see Section 2).

### 3.2. Characteristics of the Health Attitude Segments

**Figure 2:**
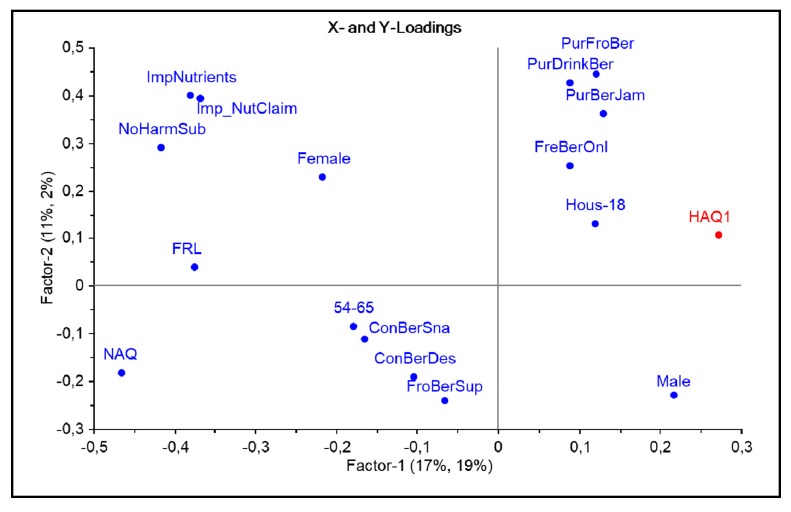
Significant attitudinal, behavioral and socio-demographic factors characterizing organic consumers in the low health attitude segment (HAQ1) in the PLS-R model. Variables projected to the right positively correlate to belonging to the (HAQ1) group; variables projected to the left negatively correlate to this group.

Discriminative characteristics of health attitude segments HAQ1 and HAQ3 were extracted by means of PLS-R modeling. The models showed 19% and 21% explained Y-variance on the first PLS component, respectively (validation variance 18% and 19%, resp.). Only variables that showed statistically significant relationships to HAQ in the jack-knifing test are reported; for an elaboration of the variables’ abbreviations, see Table 1. Figure 2 shows that respondents in the low health attitude group (HAQ1) were typically males and households with children (Hous-18), and not in the eldest age group (54–65 years old). Their berry purchases consisted in particular of convenient, long shelf life products (frozen berries, berry-based drinks, and berry jams) in addition to fresh berries, while they seldom consumed berries as a snack or as a dessert. These consumers were more often online shoppers than the other groups. Furthermore, these respondents expressed a low importance of nutrients, nutrition claims, or the absence of health-harming substances as factors in their choice of berry products. Finally, respondents in the HAQ1 group showed low natural attitudes (NAQ) and low organic and ecological attitudes (FRL) compared to the rest of the respondent sample, while they did not stand out in terms of food technology neophobia (FTNS). We may also note that none of the three countries was systematically related to low health attitude.

**Figure 3:**
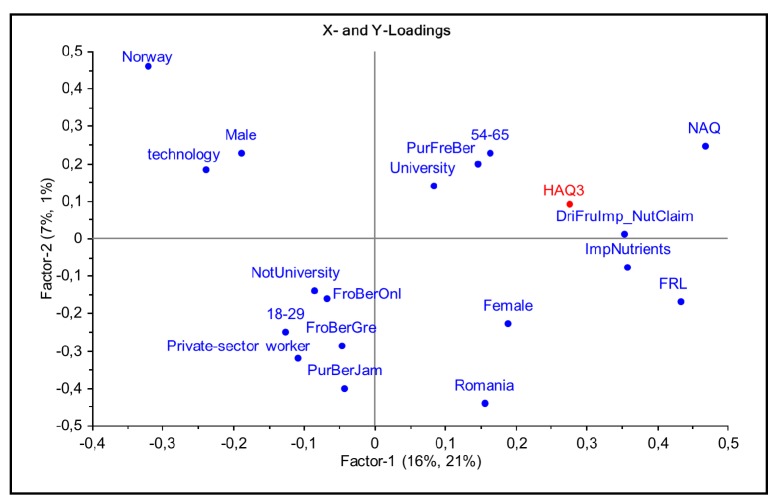
Significant attitudinal, behavioral and socio-demographic factors characterizing organic consumers in the high health attitude segment (HAQ3) in the PLSR model. Variables projected to the right positively correlate to belonging to the (HAQ3) group; variables projected to the left negatively correlate to this group.

On the opposite side of the health attitude scale, respondents in the high interest group (HAQ3) were typically females, highly educated, seldom private-sector workers, and in the 54–65 age group but not in the 18–29 age group (Figure 3). Their berry purchases consisted of fresh berries, but neither of frozen berries (neither online nor in grocery stores) nor of berry jam. These respondents expressed a high importance of nutrients content and nutrition claims as factors in their choices of berry products. Finally, respondents in the HAQ3 group showed high Natural attitudes (NAQ) and high Organic and Ecological attitudes (FRL) scores compared to the rest of the respondent sample, and rejected dried strawberries from microwave-drying technology in the choice task. These respondents were more typically from Romania and less typically from Norway.

The HAQ2 group with middle health attitude was poorly modelled (Y-calibration variance: 3%; Y-validation: 2%), indicating low systematic structure in this group’s responses. No salient socio-demographic, attitudinal, or behavioral characteristics emerged to characterize this segment (results not shown).

### 3.3. Factors Connected to Nutrient Labels Choices in Organic Consumers

**Figure 4:**
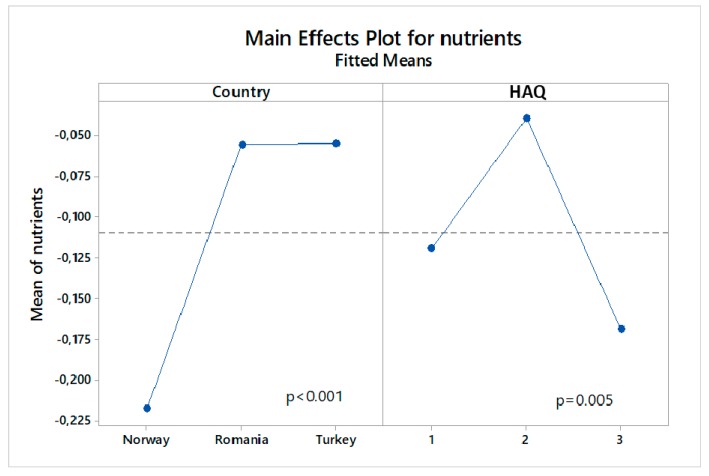
Significant main effects of (Country left) and Health attitude (HAQ score, right) on organic consumers’ choices for the “more natural nutrients (antioxidants, vitamin C, and fibers)” options in the choice task.

**Figure 5:**
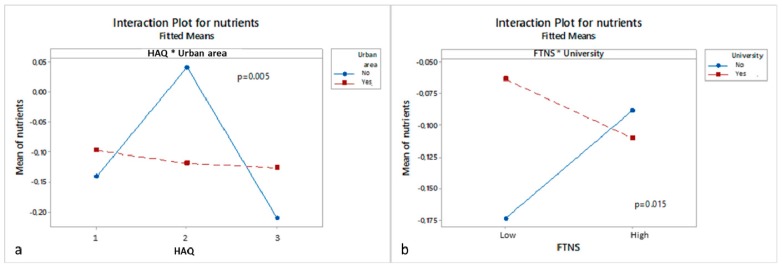
Significant interaction effects of health attitude (HAQ) * Urban area (**a**) and food technology neophobia (FTNS) * University (**b**) on organic consumers’ choices for the ‘more Natural nutrients (antioxidants, vitamin C, and fibers)’ options in the choice task.

The refined ANOVA model reveals eight factors significantly involved in main effects or interactions connected to organic consumers’ choices of nutrient labels in the choice task (Table 3). Figure 4 and Figure 5 display the size and direction of selected effects. In these plots, the *y*-axis corresponds to the variable of individual ML model estimates for design factor ‘nutrients’, which is used as the dependent variable in the ANOVA (see section Materials and Methods). Firstly, Romanian and Turkish consumers more often selected products with a “more natural nutrients” label than Norwegian consumers (Figure 4). Country also interacts with income, as in Norway high-income respondents make fewer ‘more natural nutrients’ choices than low-income respondents while no income effect is observed in Romania and Turkey (results not shown). Secondly, a two-way interaction between sex and age reveals that in younger age groups (18–41 years) females preferred packs with the ‘more natural nutrients’ label compared to males, middle-age respondents (42–53 years) responded equally across genders, while in the older age group (54–65 years) males more often chose packs with ‘more natural nutrients’ than females (results not shown). Thirdly, a significant effect of health attitude segments occurs where the HAQ2 group (middle health attitude) more frequently chose the ‘more natural nutrients’ label than HAQ1 and HAQ3 (Figure 4). Additionally, factor HAQ interacts with urban area: respondents of moderate health attitude (HAQ2 group) who live in an urban area made more choices of “more natural nutrients”-labeled products than respondents from rural areas across all three health attitude groups (Figure 5a). Fourthly, food technology neophobia attitudes interact with income (high-income respondents make fewer choices of more nutrients products than low-income respondents; results not shown) and education groups, where a university degree reverses the skepticism observed for more nutrients in respondents with technology neophobia (Figure 5b). Finally, one may note that consumption frequency of organic products was not significantly related to consumers’ nutrition label choices, neither as a main effect nor in interactions. This variable was therefore not retained in the refined model.

## 4. Discussion

Much evidence has been put forward in the literature that establishes an association between health interest and organic consumption, and shows that consumers infer healthiness properties to organic products in an “organic = healthy” assumption (see e.g., [54,70]). However, previous studies have reported somewhat conflicting results on the role of health interest as a primary driver for organic food consumption [41,69,71]. This manuscript investigated the heterogeneity in health attitudes among multicultural organic consumers, and the relationships between health attitudes and consumers’ choices for varying nutrition labels on organic dried strawberry products.

### 4.1. Organic Consumers and Health Attitudes

A simplified view of organic consumers assumes they are a fairly homogeneous segment with strong concerns to health and sustainability. Our analysis conducted on a cross-national sample of 614 consumers reveals a different reality, where large individual variations in health attitudes were observed among our respondents. On a cross-cultural level, Romanian and Turkish organic consumers showed larger health, natural and ecological interests and more often selected “more natural nutrients” labels than Norwegian consumers. One possible explanation is that the higher maturity and extent of the organic market in Norway, where organic products are available in any typical supermarket, may result in a broader range of organic consumers who are not primarily driven by health, natural, or ecological values. Specifically, previous research indicates a large variation in consumer motives for choosing organic products, including the conviction of a better taste, food safety, naturalness, quality assurance, and status signaling [63,81,82,83,84]. Interestingly, recent research has also highlighted how internal versus external personal health locus of control (i.e., the degree to which people believe that they have control over their health, as opposed to external forces beyond their control (source: Wikipedia)) moderates organic food intake [85]. While consumers with an external locus of control are misled by the label and consume more unhealthy food if marked as organic, consumers with an internal locus of control consume less [85].

The section of the pooled consumer sample into health attitude groups led to two distinct consumer profiles corresponding to the low and high health attitude groups. Corroborating previous literature, respondents in the low health attitude group (HAQ1) were often young and males [75,86,87]. They reported low natural and ecological attitudes, expressed low interest in nutrients and nutrition claims in the habitudinal questionnaire, and preferred ‘natural nutrients’ labels in the choice task. This group therefore presents high internal validity throughout the survey. Also in line with the literature, respondents in the high attitude group (HAQ3) were typically females, highly educated, in the elder age group [75,86,87]. As could be expected, these respondents reported high natural and ecological attitudes, and expressed high interest in nutrients and nutrition claims in the habitudinal questionnaire. Paradoxically, this group also preferred ‘natural nutrients’ labels in the choice task as is discussed below.

### 4.2. Nutrition Label Choices

The diverging health attitudes that we observed were related to different selections of nutrients label alternatives in the choice tasks. More specifically, a non-linear effect of health attitude was revealed, where both the low and high health attitude groups rejected the healthier label offering more nutrients, while the moderate health attitude group more often preferred this alternative. It is not surprising that HAQ1 did not consider nutrient claim as important, in line with their attitudes. According to the literature, these consumers may lack knowledge: having low nutritional knowledge and/or being unaware that claims are regulated may lead to a disbelief of the label that is seen as a pure marketing tool [88]. Corroborating the importance of knowledge, Dominick and colleagues [89] conducted a national representative survey in the US with 1000 respondents to investigate the effect of an “all-natural” label on a set of nine product categories. These authors report than being male and having too little information both decreased the likelihood of purchase.

Furthermore, we expected a clear preference for more nutrients in the HAQ3 group, which was disproved. Several explanations may be brought forward from the literature. The first one is that claims have been found to decrease tastiness, attractiveness, and naturalness perceptions [88]. More specifically, respondents in this group may have interpreted the ‘more natural nutrients’ label as a sign for ultra-processing and less naturality, which are highly in contradiction with their values. This may all the more be the case as processing technology was one of the product attributes under evaluation in the choice task, which may have primed these consumers’ skepticism for technology. Another possibility is that these consumers may not have felt in the target group for this product alternative. In a recent qualitative study, Benson and colleagues [88] identified that consumers perceive nutrition claims as mostly relevant and beneficial to specific populations presenting health issues (e.g., diabetes) or in a life phase characterized by the need for nutritional adjustments, such as dieting for weight loss, pregnancy or old age. In line with these results, Berhaupt-Glickstein and colleagues [90] found that health-risk reduction claims increased purchase intentions for green tea particularly in consumers who had modified their diet in the past year due to health concerns.

Respondents in the HAQ2 group did not express strong attitudes neither with regards to nutrients claims, nor with regards to naturality. Consequently, in the choice task, these respondents may have simply seen the indication of more nutrients as a valuable feature of the product. More research is needed to investigate whether moderate health attitudes consistently lead to an increased attractivity towards nutrient labels in other settings and product types.

To our knowledge, very few research studies have previously investigated organic consumers’ choices for organic products with nutrition labels. Aschemann-Witzel and colleagues [91] investigated the impact of combining functional food claims with organic labels on organic consumers’ product choices, and concluded that nutrition claims can be beneficial in the marketing of organic products. More specifically, these authors report that organic consumers choose organic foods with claims over those without claims, and especially so when it comes to occasional organic buyers. Their study did not investigate the role of organic consumer’s health attitude on their choices. In contrast, our data presents no evidence of the role of organic consumption frequency on consumers’ nutrient label choices, when choosing between two nutrient labels on a healthy product.

### 4.3. Limitations

In this study, the choice task investigating nutrition labels used organic dried strawberries as a case product. Effects of nutrition claims on consumer purchase decisions have been reported to be product dependent in terms of product category [85,88]. Another limitation of the study relates to the packaging design in general and to the label design in particular. Previous research has highlighted the impact of forms, colors, font types, etc. on the perception of food packaging information [92,93] including on the communication of nutrient or nutritional content [94,95]. Finally, the organic food market differs significantly across nations as a result of different geographies, agricultural policies, and public attention [50,96]. Consequently, conclusions from the current experiment may not be directly transferable to other product types and in particular to non-healthy products, other nutrient labels, and other cultures.

## 5. Conclusions

This research highlighted the heterogeneity in health attitudes among multicultural organic consumers, and revealed a non-linear relationship between health attitudes and consumers’ choices for nutrient content in organic dried strawberries. These findings underline the complexity in targeting nutrition labels to organic consumers. Three organic consumer groups of different health attitudes levels were profiled and characterized in terms of salient habitudinal, attitudinal, behavioral, and socio-demographic characteristics as well as in terms of stated nutrient content choices. This information may help organic-food marketers to better target nutrition labeling strategies at consumer segments of different health attitudes. Complementary research is recommended to test the robustness of our findings across several product categories, label designs, as well as across several cultures. Moreover, further research is needed to uncover what attitudes, motives, and knowledge drive organic consumers of different health attitudes towards different nutrition label choices.

## Figures and Tables

**Table 1:** Attitudinal and behavioral items from the consumer questionnaire.

**Variable**	**Attitudinal Items ^1^**
HAQ	The healthiness of food has little impact on my food choices (R) I am very particular about the healthiness of food I eat I eat what I like and I do not worry much about the healthiness of food (R) It is important for me that my diet is low in fat I always follow a healthy and balanced diet It is important for me that my daily diet contains a lot of vitamins and minerals The healthiness of snacks makes no difference to me (R) I do not avoid foods, even if they may raise my cholesterol (R) I do not avoid foods, even if they may be high in sugar content (R) I pay attention to the salt intake in my diet
NAQ	I try to eat foods that do not contain additives I do not care about additives in my daily diet (R) I do not eat processed foods, because I do not know what they contain I would like to eat only organically grown vegetables In my opinion, artificially flavored foods are not harmful for my health (R) In my opinion, organically grown foods are no better for my health than those grown conventionally (R)
FRL	I always buy organically grown food products if I have the opportunity I make a point of using natural or ecological food products I do not mind paying a premium for ecological products
FTNS	New food technologies are something I am uncertain about New foods are not healthier than traditional foods The benefits of new food technologies are often grossly overstated There are plenty of tasty foods around, so we do not need to use new food technologies to produce more New food technologies decrease the natural quality of food New food technologies are unlikely to have long term negative health effects (R) New food technologies give people more control over their food choices (R) New products using new food technologies can help people have a balanced diet (R) New food technologies may have long term negative environmental effects It can be risky to switch to new food technologies too quickly (R) Society should not depend heavily on technologies to solve its food problems (R) There is no sense trying out high-tech food products because the ones I eat are already good enough The media usually provides a balanced and unbiased view of new food technologies (R)
	**Behavioral Items ^2^**
PurFreBer	Purchase of berry products: _Fresh berries in the warm seasons
PurBerJam	Purchase of berry products: _Berry jams
PurFroBer	Purchase of berry products: _Frozen berries
PurDrinkBer	Purchase of berry products: _Soft drink or smoothie with berries
FreBerOnl	Where do you usually buy [fresh] berries?_Online grocery store
FroBerGre	Where do you usually buy [frozen] berries?_Greengrocer’s
FroBerSup	Where do you usually buy [frozen] berries?_Supermarket/hypermarket
FroBerOnl	Where do you usually buy [frozen] berries?_Online grocery store
Imp_NutClaim	When you buy dried fruits or dried berries, how important are the following characteristics for you?_Nutrition Claim
ImpNutrients	When you buy food products based on dried fruits or on dried berries, how important are the following characteristics for you?_Nutrients
NoHarmSub	When you buy food products based on dried fruits or on dried berries, how important are the following characteristics for you?_No health-harming substances
ConBerSna	In which context do you consume dried berries?_Snacking
ConBerDes	In which context do you consume dried berries?_Dessert

HAQ: health attitudes from the Health and Taste Attitude Questionnaire [75]; NAQ: natural attitudes from the Health and Taste Attitude Questionnaire [75]; FRL: ecological attitudes from the Food-Related Lifestyle (FRL) scale [76]; FTNS: attitude towards new food technology from the Food Technology Neophobia Scale (FTNS) [77]. ^1^ (R) indicates reversed items for the computation of attitudinal scores. ^2^ Only the attitudinal and behavioral items relevant for the results section are shown.

**Table 2:** Attitudinal characteristics of the consumers in Norway, Romania, and Turkey and pooled sample, and attitudinal group thresholds for further analysis

Attitudes	Norway (*n* = 204)	Romania (*n* = 206)	Turkey (*n* = 204)	Pooled (*n* = 614)	Attitudinal Groups (*n* = 614)
	Mean (SD)	Mean (SD)	Mean (SD)	Mean (SD)	[Mean Score Range], Group Size
Health attitude (HAQ)	4.64 ^a^ (0.79)	4.94 ^b^ (0.93)	5.07 ^b^ (0.93)	4.89 (1.00)	HAQ1: [1.6–4.2], 174 HAQ2: [4.3–5.4], 265 HAQ3: [5.5–7.0], 173
Natural attitude (NAQ)	4.44 ^a^ (1.16)	5.26 ^b^ (0.96)	5.31 ^b^ (1.03)	5.00 (1.12)	Low: [1.0–5.0], 314 High: [5.1–7.0], 298
Ecological attitude (FRL)	4.15 ^a^ (1.40)	5.64 ^b^ (1.11)	5.35 ^c^ (1.25)	5.05 (1.42)	Low: [1.0–5.0], 318 High: [5.1–7.0], 294
Food technology neophobia scale (FTNS)	4.18 ^a^ (0.91)	4.60 ^b^ (0.78)	4.43 ^c^ (0.91)	4.40 (0.89)	Low: [1.2–4.3], 324 High: [4.4–7.0], 288

^a,b,c^ Significant differences based on chi-squared and Pearson chi-squared tests. Same letter indicates that there is no statistical significant difference at a 5% level.

**Table 3:** Analysis of variance (ANOVA) model for nutrient label choices

Source	DF	Adj SS	Adj MS	F-Value	*p*-Value
Country	2	1.9831	0.991554	15.22	0.000
Sex	1	0.0001	0.000095	0.00	0.970
Age	3	0.1052	0.035055	0.54	0.656
HAQ	2	0.6907	0.345329	5.30	0.005
FTNS	1	0.0275	0.027495	0.42	0.516
Income	3	0.3056	0.101861	1.56	0.197
Urban area	1	0.0063	0.006307	0.10	0.756
University	1	0.1661	0.166091	2.55	0.111
Country × Income	6	1.0368	0.172796	2.65	0.015
Sex × Age	3	0.5851	0.195020	2.99	0.030
HAQ × Urban area	2	0.7095	0.354760	5.45	0.005
FTNS × Income	3	0.6797	0.226572	3.48	0.016
FTNS × University	1	0.3869	0.386895	5.94	0.015
Error	535	34.8531	0.065146		
Lack-of-Fit	367	28.0640	0.076469	1.89	0.000
Pure Error	168	6.7891	0.040411		
Total	564	42.2876			

HAQ: Health attitude score, from the Health and Taste Attitude Questionnaire [74]. FTNS: Food technology neophobia score, from the Food Technology Neophobia Scale (FTNS) [76]. DF: degrees of freedom; Adj SS: adjusted sum of squares; Adj MS: adjusted mean squares; F-value: the test statistic used to determine whether the term is associated with the response; *p*-value: a probability that measures the evidence against the null hypothesis (source: https://support.minitab.com/).

## Supplementary Materials

The following are available online at https://www.mdpi.com/2072-6643/11/12/2951/s1, Figure S1: An example of choice set (English translation).
