# Supplementary material for: Organic Consumer Choices for Nutrient Labels on Dried Strawberries among Different Health Attitude Segments in Norway, Romania, and Turkey
Source: Nutrients. 2019 Dec 4;11(12):2951. doi: 10.3390/nu11122951 (PMC6950596; doi:10.3390/nu11122951)

Figure S1: An example of choice set (English translation).

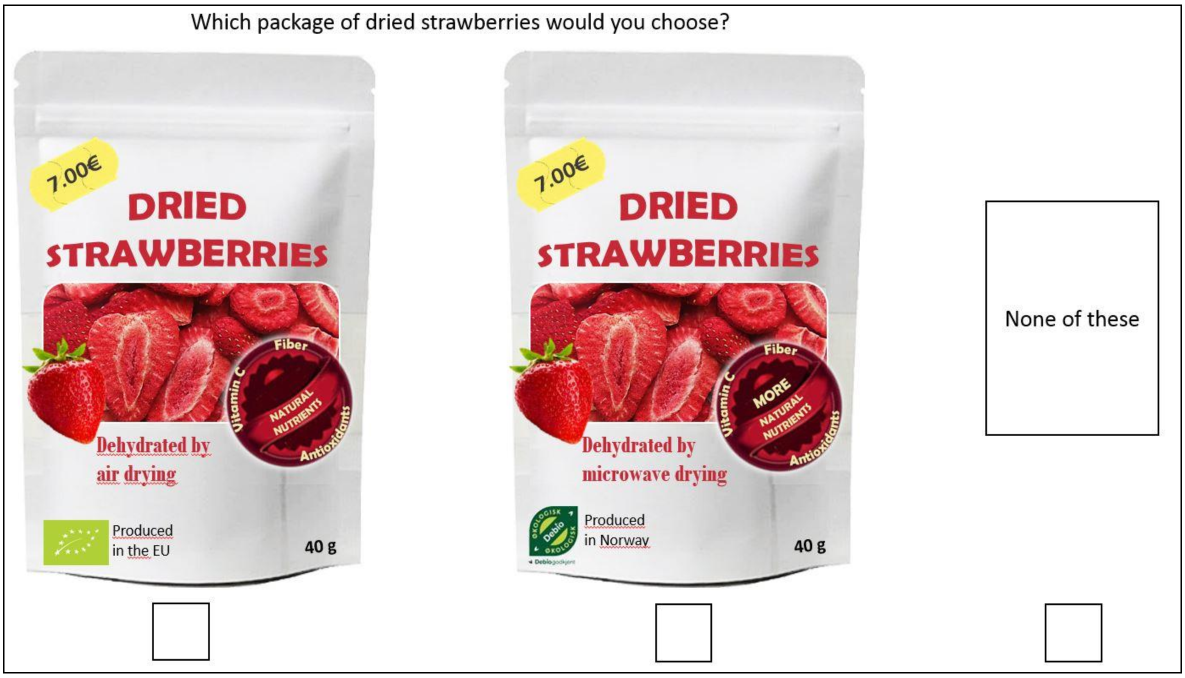

Supplement: Supplementary file 1 [file nutrients-11-02951-s001.pdf]
